# Vestibular function in cochlear implant users

**DOI:** 10.1016/S1808-8694(15)31100-9

**Published:** 2015-10-19

**Authors:** Ariane Solci Bonucci, Orozimbo Alves Costa Filho, Luciane Domingues Figueiredo Mariotto, Regina Célia Bortoleto Amantini, Kátia de Freitas Alvarenga

**Affiliations:** 1 Speech and hearing specialist - Head-Facial Anomalies Rehabilitation Hospital - University of São Paulo HRAC/USP, Bauru/SP; 2 Full Professor - University of São Paulo, Coordinator - Head-Facial Anomalies Rehabilitation Hospital - University of São Paulo HRAC/USP, Bauru/SP; 3 Specialist in audiology, speech and hearing therapist - Speech and Hearing Clinic - Dentistry School - Bauru University of São Paulo, FOB/USP, Bauru/SP; 4 PhD. Speech and hearing specialist - Head-Facial Anomalies Rehabilitation Hospital - University of São Paulo HRAC/USP, Bauru/SP; 5 PhD. Professor of speech and hearing therapy - Speech and Hearing Clinic - Dentistry School - Bauru University of São Paulo, FOB/USP, Bauru/SP. Hospital de Reabilitação de Anomalias Craniofaciais da Universidade de São Paulo (HRAC/USP) - Centro de Pesquisas Audiológicas (CPA)

**Keywords:** cochlear implant, vertigo, vestibule

## Abstract

Balance alterations in the postoperative of cochlear implant surgeries varies from 31 to 75%.

**Aim:**

to analyze vestibular function in the pre and postoperative periods of cochlear implanted individuals.

**Materials and methods:**

the vestibular function was assessed, through electronystagmography, in 38 patients, in the pre and postoperative of cochlear implant procedures. The main complaint of unbalance reported by patients was dizziness, followed by postural vertigo and non-postural vertigo.

**Results:**

13% of the patients did not show any balance disorder following cochlear implant surgery and just 5% showed symptoms worsening. 13 % of the patients showed an improvement, and this could be related to the vestibular compensation phenomenon and to electric stimulation. However, it was observed, in the caloric responses, a worsening in the vestibular system function, for both implanted and non-implanted ears. Thus, there is no evidence of more damage to the implanted ear.

**Conclusion:**

the study showed that cochlear implant surgeries could injure the vestibular system in both ears. However, the vestibular symptoms take place in a smaller proportion, and can improve after cochlear implant surgery.

## INTRODUCTION

Because of the very anatomic proximity between the auditory and vestibular systems, and their embryologic and physiologic interactions, they may be simultaneously involved in some bodily dysfunctions. This involvement is more frequent in peripheral alterations than in central ones^1^.

Studies performed^2^^,^^5^, ^6^, ^7^, ^8^, ^9^ in individuals with cochlear implant (CI) with the goal of assessing the vestibular system presented variability in their results, both in terms of symptoms as well as the alterations seen in the postoperative evaluation.

Among the complications caused by implanting electrodes in the cochlea, we can mention changes in the normal fluid homeostasis of the inner ear, trauma in vestibular sensorial structures or surgery-induced inflammation resulting in fibrosis or loss of hair cells. Together with all of this, the electrical stimulation of the cochlear implant can cause pathologic changes in the inner ear as a subsequent dysfunction of structures, resulting in vestibular alterations^2^, ^3^, ^4^.

In the specific literature we see that balance disorders in the CI postoperative period vary between 31 and 75%^5^, ^6^, ^7^, ^8^, ^9^. In relation to vestibular tests, although some studies have found an improvement in the caloric test after CI surgery,^10^ it can not be reliable to predict post-operative vestibular symptoms^2^^,^^6^. Another important information found in the caloric test was that the changes that occurred in the implanted ear were followed by similar changes that happened in the non-implanted ear^2^.

Recent investigations have found a correlation between the vestibular exam results after CI surgery and the vertigo variables in the preoperative, age at surgery and time of hearing loss^6^. However, the same correlation was not seen with the variables: etiology, implanted ear, age at surgery and implant type^9^.

An important piece of information that must be considered in the evaluations is that since many of these patients already had vestibular alterations before CI surgery, the effects of CI in the vestibular system can be underestimated^11^. The incidence of patients with vestibular problems associated with cochlear implant who developed benign positional vertigo (BPV) after surgery is of 159/100,000 per year, and this ratio is higher than that in the general population (ratio of 64/100,000 per year)^12^.

In the specific literature, we found only one study that reported a significant improvement in the objective and subjective vestibular function evaluations after surgery and CI activation^2^.

## GOAL

The present study aims at analyzing the vestibular function in the pre and post operative stages of cochlear implant surgery, by means of the caloric test.

## MATERIALS AND METHODS

This investigation was approved by the Ethics Committee - # 394/2006-SVAPEPE-CEP.

### Sample selection

Initially, we selected the individuals who underwent preoperative vestibular evaluation, encompassing all the tests proposed by Mangabeira Albernaz et al. (1981)^13^. Thus, we took off the study those individuals who presented incomplete vestibular tests because of age in the preoperative stage and those who presented clear signs of central disorder in the positional nystagmus tests, pendulum tracking, optokinetic nystagmus, spontaneous nystagmus with the eyes open and/or decreasing pendulum rotational test.

### Sample

The sample was made up of 38 individuals submitted to a multichannel cochlear implant surgery, with surgical age between 4 and 62 years (30.65±16.32 years).

### Assessment process

The assessment was carried out in the CI pre and postoperative periods. The postoperative stage was analyzed considering two moments: hospital admittance and after device activation (during the time the patient used the cochlear implant).

### Vestibular symptoms analysis

Data related to vestibular symptoms (dizziness, postural and non-postural vertigo) in the pre and postoperative (hospital) stages were obtained from the patient's records. And in the postoperative stage, an interview with objective questions was carried out.

### Caloric test

The warm water test was used to assess the vestibular function, because it is the procedure that allows checking the ears separately. For analysis we considered the post-caloric nystagmus.

The caloric test was carried out with water in the preoperative, with the OC-214 model oto-calorimeter, and air in the postoperative, with the AR-314 oto-calorimeter, both from Berger. In the water stimulation, the temperatures used were 44$\underline{\text{o}}$C and 30$\underline{\text{o}}$C and 40s for stimulation time in each ear. There was a 5 minute interval between each application. The chair was tilted in 45$\underline{\text{o}}$ backward, allowing a 45$\underline{\text{o}}$ tilt of the individual's torso in relation to the horizontal plane. The responses were recorded first with the eyes closed after stimulation and then with the eyes open in order to check for eye inhibitory effects during 10s.

We investigated spontaneous and post-caloric nystagmus in order to check for possible interference they could cause on the caloric test results. In order to carry on the tests with the eyes closed, we talked to the individual in order to cause cortical relaxing.

The caloric test was analyzed as to the values of the slow component angular velocity (SCAV), classified in the following way (Chart 1).

**Chart 1:** Classification according to the VACL values

	4 to 12 years (water) 14	Adults(water) 12	Adults/children (air) 15
Normoreflexia	from 6.9 to 51.2$\underline{\text{o}}$/s	from 3 to 50$\underline{\text{o}}$/s	from 2 to 28$\underline{\text{o}}$/S
Hyporeflexia	> 6.9$\underline{\text{o}}$/S	> 3$\underline{\text{o}}$/S	> 2$\underline{\text{o}}$/S
Areflexia	0	0	0
Hyperreflexia	< 51.2$\underline{\text{o}}$/s	>50$\underline{\text{o}}$/s	< 28$\underline{\text{o}}$/S

### Results analysis

The data obtained was organized in Tables and Figures, using descriptive statistics. For comparison and correlation of the findings with the variables studied, we used the Chi-square, Kruskal-Wallis ANOVA, McNemar tests. We considered p<0.05 as a significant value.

It is worth stressing that we did not use the slow component angular velocity numeric values (absolute values) and that of directional and/or labyrinth preponderance (relative values) when analyzing the results from this study, because the caloric test was carried out with different methods for stimulation: water in the preoperative stage and air in the cochlear implant postoperative stage. Thus, the analysis criteria were the conclusive results of the caloric test, according to what is shown on Chart 1.

## RESULTS

Table 1 shows the demographic data of the 38 patients evaluated.

**Table 1:** Demographics on the 38 patients evaluated.

Characteristics of this series of 38 patients	
Age at the CI surgery	N (%)
Age ≤12 years	7 (18)
Age >12 years	31 (82)
Gender	
Female	15 (39)
Male	23 (61)
Type of insertion	
Total	36 (95)
Partial	2 (5)
CI Type	
Nucleus 22	11 (26)
Nucleus 24K	6 (14)
Med El	23 (53)
Clarion	3(7)
Implanted Ear	
RE	21 (55)
LE	17 (45)
HL Etiology	
Congenital	4 (10)
Ototoxic	3 (8)
Unknown	10 (26)
Hereditary	2 (5)
Meningitis	9 (24)
Head Injury	9 (24)
Others	1 (3)

CI: cochlear implant; HL: hearing loss

RE: right ear; LE: left ear

## Vestibular symptoms

We have noticed that, of the 38 patients evaluated, 58% (22) complained of unbalance at the presurgical stage.

Analyzing the hospital stay period, immediately after CI implant, of the 38 patients, we did not find information on the vestibular symptoms of seven patients and they did not report subsequent treatments. Thus, considering 31 patients, 81% (25) did not complain of unbalance in the preoperative and reported that the symptoms remained in the hospital stay period and during the month that preceded the activation.

In the post-surgical stage, considering the period of cochlear implant use, we noticed that of the 31 patients, 77% (24) did not have it; and 3% (1) had vestibular complaints, both kept the symptoms, 17% (5) did not have more vestibular symptoms, and only 3% (1) started to complain of symptoms after cochlear implant surgery.

The type of vestibular symptom the patients had in the pre and post-surgical stages is shown on Table 2.

**Table 2:** Vestibular symptoms the patients had in the CI pre and postoperative.

Symptoms	Pre (%)	Post (%)
Dizziness	55	61
Positional vertigo	36	28
Non-positional vertigo	9	11

**Figure 1:**
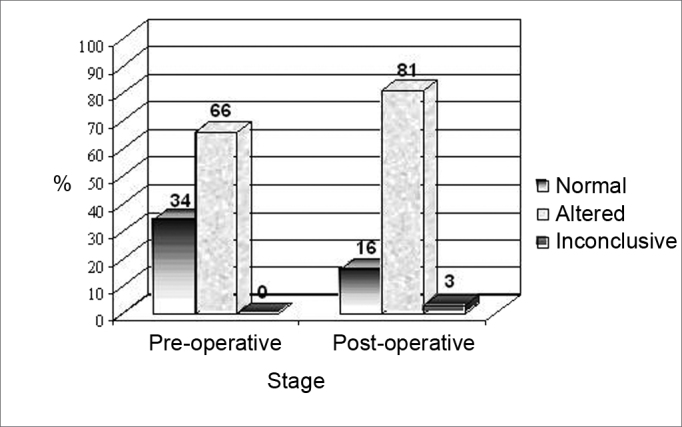
Results from the caloric test in the pre and postoperative stages in the implanted ear.

Figure 1 shows the results from the caloric test in the pre and post-caloric stages in the implanted ear.

In the comparative analysis of post-caloric nystagmus in the implanted ear before and after surgery, we noticed that 66% (25) of the patients had altered post-caloric nystagmus (hyperreflexia, hyporeflexia and areflexia) and 34% (13) were normal in the preoperative stage. On the other hand, in the postoperative stage, 81% (31) of the patients had altered post-caloric nystagmus, only 16% (6) were normal and 3% (1) had inconclusive results.

**Figure 2:**
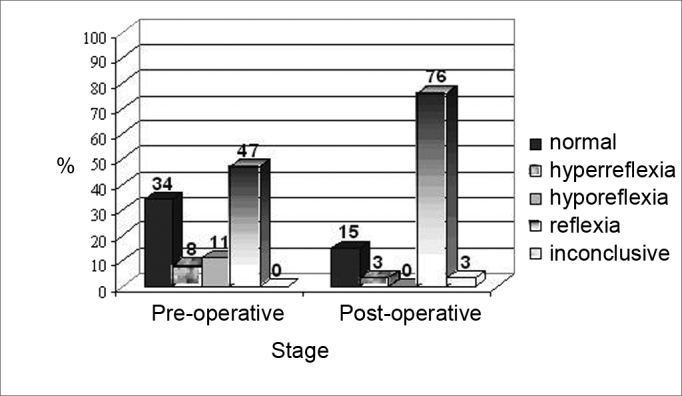
Results from the caloric test in the pre and postoperative stages in the implanted ear.

Figure 2 shows the results of the caloric test, considering normoreflexia, hyporeflexia, areflexia and hyperreflexia for the implanted ear, in the pre and postoperative stages.

Of the 13 individuals with normal preoperative nystagmus, 31% (4) were normal, 8% (1) had inconclusive results and 61% (8) started having alterations, 87% (7) had areflexia and 13% (1) hyporeflexia. On the other hand, 10% (4) of the individuals who had hyporeflexia started having areflexia.

Considering that, in some patients there was a change in the caloric test result after cochlear implant surgery (improved, maintained the same or worsen), and we noticed a correlation between the occurrence of change in the following variables: gender, age at the time of surgery and duration of hearing impairment (Table 3).

**Table 3:** Results from the statistical analysis used to check the correlation between the occurrence of change in the post-caloric test and the pre and post-operative stages in the implanted ear, with the variables gender, age at the time of surgery and hearing loss duration.

CHANGE IN THE CALORIC TEST RESULT - implanted ear	
Gender	0.17
Age at the time of surgery	0.64
Hearing loss duration	0.37

p£0.05: statistically significant.

**Figure 3:**
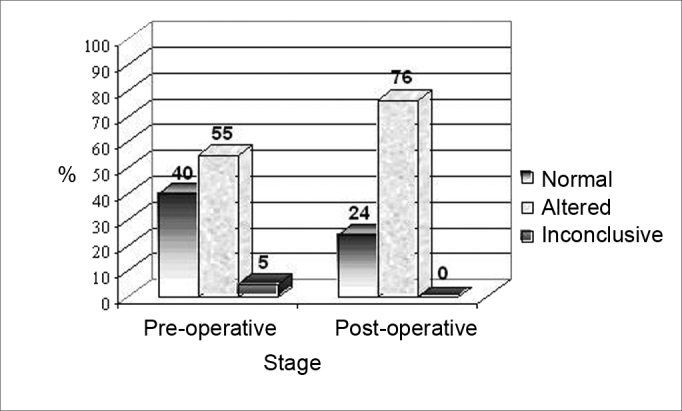
Results from the caloric test in the pre and postoperative stages in the non-implanted ear.

**Figure 4:**
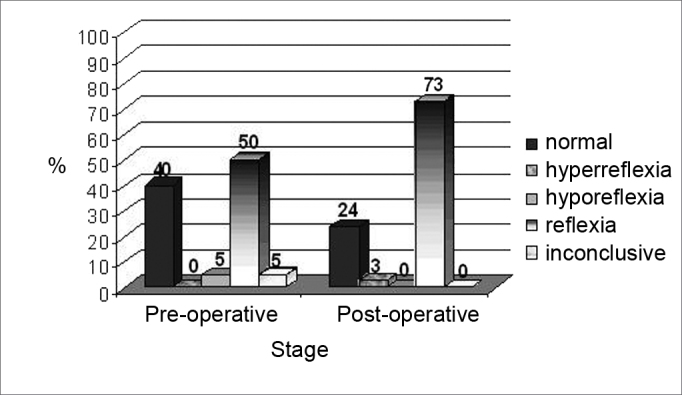
Results from the caloric test in the pre and postoperative stages, in the non-implanted ear.

Figure 3, Figure 4 present the caloric test results, characterizing the changes seen for the non-implanted ear.

In the comparative analysis of the post-caloric nystagmus of the non-implanted ear in the pre and post-surgical stages, we observed that 55% of the patients (21) presented altered post-caloric nystagmus (hyperreflexia, hyporeflexia and areflexia) and 40% (15) were normal at the pre-surgical stage. On the other hand, in the postoperative, 76% (29) of the patients presented altered post-caloric nystagmus and 24% (9) were normal.

Of the 15 individuals with normal preoperative nystagmus, 47% (7) maintained normal, 53% (8) started to have an alteration: 12% (1) hyperreflexia and 88% (7) areflexia. On the other hand, 5% (2) of the individuals who had hyporeflexia changed to areflexia.

For the statistical analysis of the results obtained in the vestibular tests and its correlation with the implanted and non-implanted ear, the results obtained in the vestibular tests in the pre and post surgical stages were divided in three groups: no alteration in the results, better or worse results, considering normoreflexia, hyporeflexia, areflexia and hyperreflexia. In checking whether or not there was a greater change trend in the vestibular function of the implanted ear when compared to the non-implanted ear, we did not find any statistically significant difference, p=0.446.

## DISCUSSION

In the present investigation, we assessed 38 patients submitted to multichannel cochlear implant surgery, with the Nucleus 22, Nucleus 24K, Med El and Clarion devices.

In this study, the main complaint of unbalance presented by the patients was dizziness, followed by positional vertigo and later by non-positional vertigo, both in the pre and postoperative periods.

Vestibular symptoms in the preoperative of some of the patients evaluated (58%) is associated with the etiology of hearing impairment, having seen that, in some, the unbalance is part of the clinical status (Table 1). Thus, the analysis of the cochlear implant surgery impact on the vestibular apparatus is impaired, because the unbalance is a common complaint in many patients, according to the literature studied^11^.

Nonetheless, it was possible to see that 17% of the patients stopped complaining of unbalance after the CI surgery, and only 3% of the 31 patients reported worsening, and the other ones did not notice changes in the symptoms or never had unbalance problems. The occurrence of unbalance after surgery (5%) was less than what is described in the literature, and varied between 31 and 75%^5^, ^6^, ^7^, ^8^, ^9^. Another important point is the fact that 17% of the patients reported improvements in their vestibular symptoms, and such possibility has been already described as being associated with the phenomenon of vestibular compensation and electrical stimulation^2^.

Considering the results obtained in the caloric test carried out in the implanted ear, it was possible to see that there was a worsening in vestibular system function, characterized by the reduction of individuals with normal post-caloric nystagmus, the symptoms of hyporeflexia and the increase in vestibular areflexia (absence of post-caloric nystagmus) in the post-surgical stage (Figure 1, Figure 2). The alteration in the vestibular system was described in the literature as one of the possible complications caused by implanting electrodes in the cochlea^2^, ^3^, ^4^, and such finding has been confirmed in this study.

A similar finding was observed in the non-implanted ear, with a worsening of the vestibular function in some patients (Figure 3, Figure 4), and the changes observed were similar between the ears, there was no trend of a greater involvement of the implanted ear (p=0.446). Prior studies described a similar finding^2^^,^^6^^,^^10^, however, the reason behind this phenomenon is still unknown.

We hereby stress that, although in CI pre and postoperative stages different methods of caloric stimulation were used (water and air, respectively), this fact did not interfere in the results, because the data analysis was carried out by means of the final result of the caloric test presented in Chart 1 (normoreflexia, areflexia, hyporeflexia or hyperreflexia) and not by the absolute value of the angular velocity of the slow component obtained in the stimulations.

We did not find any correlation among the variables: gender and age at the time of surgery, and duration of hearing loss with the caloric test results in the implanted ear (Table 2). In the literature there is no consensus as to the influence of these variables in the postoperative diagnosis associated with the vestibular function^6^^,^^9^.

In the present investigation, we noticed through the objective evaluation (caloric test) that the vestibular system may be compromised by the cochlear implant surgery. Nonetheless, there is a mismatch between the occurrence of post-caloric nystagmus and the subjective complaint of unbalance that proved to be better after cochlear implant surgery^2^^,^^6^. As previously discussed, the reduction in postoperative vestibular symptoms may be associated with the electrical stimulation and the vestibular compensation phenomenon, keeping the patient from presenting or presenting minimum unbalance complaints if compared to the extent of the involvement.

Thus, in the cochlear implant centers, it is fundamentally important that within the multidisciplinary team, the speech and hearing therapy should act not only in the vestibular diagnosis process, but also in the follow up of the patient by means of vestibular rehabilitation education.

Setting up a vestibular rehabilitation program helps in a more effective occurrence of the compensation phenomenon, with consequent reduction in the symptoms of unbalance, which will positively reflect on the quality of life of an individual with multichannel cochlear implant.

## CONCLUSION

The present investigation has shown that the cochlear implant surgery may compromise the vestibular system, not only in the implanted ear, but also in the non-implanted ear, with predominance of the post-caloric nystagmus areflexia. However, the vestibular symptoms happen in a lesser proportion, and there may be an improvement in unbalance after cochlear implant surgery.
